# GO/CNT−OH/Nafion Nanocomposite Humidity Sensor Based on the LC Wireless Method

**DOI:** 10.3390/nano13131925

**Published:** 2023-06-24

**Authors:** Chengkai Wang, Chunxiao Jiao, Meng Wang, Jinghong Pan, Qi Wang

**Affiliations:** College of Sciences, Northeastern University, Shenyang 110819, China; 2100178@stu.neu.edu.cn (C.W.); jiaochunxiao@stumail.neu.edu.cn (C.J.); winnie@stumail.neu.edu.cn (M.W.); 2100169@stu.neu.edu.cn (J.P.)

**Keywords:** LC resonant sensors, humidity sensor, GO/CNT−OH/Nafion nanocomposites, highest frequency variation, resonant frequency

## Abstract

In recent years, LC resonant sensors have gained widespread attention for their extensive applications in industries such as pharmaceutical storage and food transportation. A wireless passive sensor with a good sensing performance is proposed based on a GO/CNT−OH/Nafion nanocomposite. The sensor was fabricated via inkjet printing technology, and the surface morphology of the GO/CNT−OH/Nafion nanocomposite was characterized by SEM measurement. It is found that the MWCNTs support the GO layer and the hydrophobic chains of Nafion interact with the hydrophobic layer of GO, resulting in a larger cavity and hydrophilic surface of the entire material. This structure well reflects the fact that the mixing of MWCNTs and Nafion provides the entire material with a stronger water absorption. The experimental study shows that the proposed humidity sensor has a frequency variation of 103 kHz/%RH at low humidity (30–60% RH) and a sensitivity of 931 kHz/%RH at high humidity (60–95% RH), while the sensitivity value from 30–95% RH is 547 kHz/% RH. The response time and recovery time are 110 s and 115 s, respectively. In addition, the tests showed that the GO/CNT−OH/Nafion nanocomposite applied to the humidity sensor had a maximum humidity hysteresis of about 3% RH at 30–95% RH, the resonant frequency remained basically unchanged after 50 h of testing, and the whole sensor possessed a good stability. After conducting several repeated experiments, it was found that the resonant frequency error of the whole sensor was low and did not affect the overall sensing test, which proved the reproducible preparation of the sensor. Finally, the humidity-sensing mechanism of the proposed sensor was analyzed in this paper, and it was found that GO enhanced the hygroscopic properties of GO/CNT−OH/Nafion nanocomposite when it was supported by MWCNT-OH and included uniformly dispersed Nafion. Therefore, our proposed humidity sensor is suitable for humidity detection above 30% RH in both sealed and open environments.

## 1. Introduction

Humidity detection is widely applied in the food industry, pharmaceutical storage, food transportation, and other fields [1,2,3,4]. Therefore, humidity sensors, as the core of humidity detection, should have a good linear response, high sensitivity, wide range of humidity detection, fast response and recovery performance, ideal reversibility, low cost, and long-term stability [5]. For example, V. Manikandan provided a comparison of the stability tests related to the material under corrosive conditions in the preparation of Li–NiFe_2_O_4_ nanomaterials [6]. At present, the main humidity sensors are capacitive, resistive, surface acoustic wave (SAW), and LC resonance type [7,8,9]. Resistive sensors show a good performance in the medium humidity range, but their response is weak at a lower humidity. In addition, they also encounter problems with size and circuit integration [10,11,12]. The fabrication processes of SAW devices and their required circuits are often complex and expensive [13,14]. In contrast, capacitive and LC resonant sensors that operate with a varying capacitance are widely used due to their advantages such as good linearity, high sensitivity, low signal processing circuit complexity, and low power consumption [15]. The use of capacitive sensors for humidity testing is currently widespread; for example, the capacitive humidity sensors with different pore sizes based on nanoporous alumina manufactured by M. A. Mir and the capacitive humidity sensors with freestanding bendable porous SiO_2_ manufactured by Soobin Park exhibit a good performance in humidity detection [16,17]. Compared with capacitive humidity sensors, LC resonance sensors could measure the parameter of interest wirelessly, making it an ideal choice for detecting humidity in wirelessly connected environments. For example, they can detect humidity in sealed food packaging bags, and compared with traditional active sensors, they have low manufacturing costs, simple structure, long theoretical service life, and are more conducive to building a compact, effective, and cost-effective distributed sensor network that belongs to the Internet of Things. Therefore, LC resonant sensors are an ideal choice for sealed and non-contact measurement environments [18,19,20,21]. Wang et al. fabricated a printed LC humidity sensor label and experimentally evaluated the sensitivity of the label at 1.1 kHz/% RH [22]. Feng, Y. constructed a PEL-based humidity sensor, and after experimental testing, the response time and recovery time of the sensor at 30–80% RH were 240 s and 360 s, respectively [23]. Ming-Zhu Xie et al. produce paper-based inductive sensors with a sensitivity of 140 kHz/%RH [24]. According to the above reports, certain performance indicators, including a limited detection range, response, and recovery time and complex manufacturing process, have limited the application of LC humidity sensors. A comparison of other previous work is given in Table 1. The sensing-performance characteristics of some of the sensors mentioned above are not ideal and need to be improved.

Inkjet printing technology is a non-contact printing technology that can control the production of products through computer automation. It has the advantages of a low cost, no contact with the substrate, simple molding methods, and material saving, and it enables the easier patterning of electrode materials on flexible substrates. Therefore, it has become the mainstream process for the preparation of flexible electronic devices [25,26,27].

Graphene oxide (GO), a major derivative of graphene, facilitates the formation of thin films through solution-based fabrication processes due to its functional groups (carboxyl, hydroxyl, and epoxy) which decorate the surface of the graphene honeycomb structure. These functional groups can increase the hydrophilicity of GO, thereby enhancing its sensitivity to water molecules. Therefore, graphene oxide has attracted much attention in the field of humidity sensing [28,29,30]. Furthermore, due to the large surface-to-volume ratio caused by the porous structure, weak bonding ability with hydroxyl groups in water molecules, and dense pores, multi-walled carbon nanotubes (MWCNTs) exhibit a capillary effect on humidity, thus generating a capacitive response [31,32,33,34,35]. Hydroxylated MWCNTs contain hydroxyl groups, which can form hydrogen bonds with water, resulting in an improved overall water-absorption performance. In addition, Nafion possesses hydrophilic sulfonic acid terminal groups, which can produce better humidity-sensitive properties and improve long-term stability and linearity [36,37]. Therefore, the combination of these three materials may be an applicable combination that may produce a better sensing performance. Hitherto, no one has reported the application of GO/CNT−OH/Nafion nanocomposites for humidity sensing.

This paper proposes an LC wireless passive hygrometer based on a GO/CNT−OH/Nafion sensing material. The device is fabricated by inkjet printing technology. Experiments are carried out to demonstrate the sensing performance of this humidity sensor at 30–95% RH, and the sensing mechanism of the proposed sensor in different humidity environments is also clarified through analysis. The technology is simple and can effectively reduce the manufacturing cost of the sensor and provide the sensor with a high sensitivity, good performance, and easy recovery.

## 2. Experiment

### 2.1. Materials

Graphene oxide (GO) solution (2 mg/mL) was provided by Suzhou Tanfeng Graphene Technology Co. Nafion solution (5 wt%) was obtained from DuPont company (Wilmington, DE, USA). Hydroxylated multi-walled carbon nano-tubes (>95%, 5–12 nm) were obtained from Macklin Reagents. Commercially available surface-treated PET films (with a thickness of 125 μm) were utilized, along with spray-printing silver nanoparticle conductive ink (CON-INK550) (silver powder particle size of 50 nm, containing 30–40 wt% silver, square resistance of 1~10 mΩ/□/mil, viscosity in 5~12 cP adjustable).

### 2.2. Sensor Fabrication and Design

**Figure 1:**
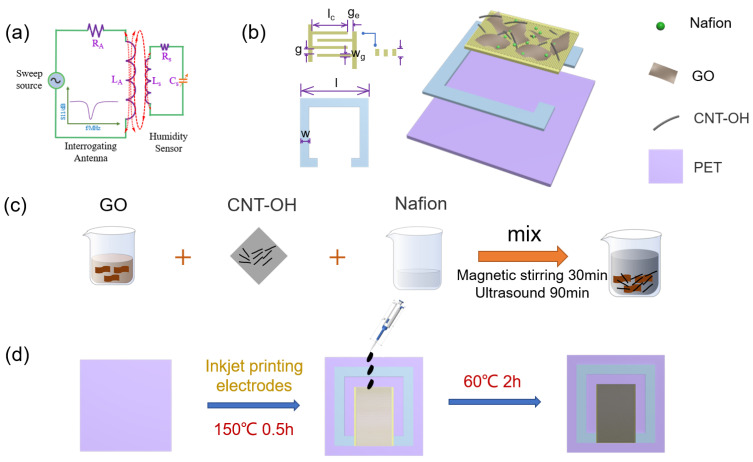
(**a**) Equivalent circuit of LC humidity sensor; (**b**) image of the designed GO/CNT−OH/Nafion nanocomposite humidity sensor; (**c**) preparation process of GO/MWCNT/Nafion nanocomposites; (**d**) preparation process of the device.

The operating mechanism of the LC-type sensor can be described as a resonant circuit, as shown in Figure 1a. As demonstrated theoretically, the resonant frequency of the humidity sensor is as follows.

(1) $$f=\frac{1}{2\pi \sqrt{L_{s}C_{s}}}$$

where *f* represents the resonant frequency of the humidity sensor, and *L_s_* and *C_s_* represent the capacitance and inductance of the humidity sensor, respectively. Due to the inductive coupling between the interrogation antenna excited by a scanning source with a wide frequency range and the humidity sensor, a resonance point (RP) can be generated; the resonance frequency of the humidity sensor can be measured wirelessly through electronic equipment such as a network analyzer. The sensitivity of *L_s_* to humidity is zero; thus, the resonant frequency only varies with the changes in the relative permittivity (*ε_r_*) in *C_s_*. The value of *ε_r_* is mainly determined by the ratio of the sensing material to the humidity. Therefore, the humidity in the surrounding environment can be detected wirelessly through the resonant frequency of the humidity sensor.

According to Kirchhoff’s law, the input impedance (*Z_in_*) of the sensor can be expressed as:

(2) $$Z_{in}=R_{a}+j\omega L_{a}+\frac{\omega^{2}M^{2}}{R_{s}+j(\omega L_{s}-1/\omega C_{s})}$$

(3) $$M=k\sqrt{L_{a}L_{s}}$$

where ω is the angular frequency of the sensor, *M* is the mutual inductance of *L_a_* and *L_s_*, and *k* is the coupling coefficient. In addition, fluctuations in the relative humidity change the S_11_ reflection coefficient by changing the input impedance *Z_in_* as:

(4) $$S_{11}=\left.\frac{Z_{in}-Z_{0}}{Z_{in}+Z_{0}}\right|Z_{0}=50\Omega$$

*Z_0_* is the interface impedance value of the network analyzer. Since the sensitivity of *L_s_* to humidity is zero and *R_a_* and *L_a_* are constant, the resonant frequency varies only with the relative capacitance (*ε_r_*) of *C_s_*. The value of *ε_r_* is mainly determined by the ratio of the sensing material to humidity. Therefore, it can be seen from Figure 1a that the humidity of the surrounding environment can be monitored by the negative peak of the *S*_11_-frequency.

As shown in Figure 1b, the humidity sensor was designed on a PET substrate (length × width × thickness: 50 mm × 50 mm × 0.125 mm). Inkjet printing technology was used to deposit nano-silver ink on this substrate. As illustrated in the figure, the number of IDEs is 60 (the number of pairs of forked finger electrodes is 30 pairs); *l* and *w* indicate the side length and width of the inductor, respectively; *w*_g_ denotes the width of the interdigital electrode (IDE); *g* is the gap between the interdigital fingers; *g*_e_ is the distance between the two electrodes; *l*_c_ is the length of the electrodes; and *h* is the thickness of the IDE and the number of IDEs (see Table 2 for specific parameters).

As shown in Figure 1c, taking GO/CNT−OH/Nafion as an example, the prepared Nafion solution was first diluted to 0.5 wt%. Then, 3 mL of a GO aqueous dispersion was added to a small 5-mL beaker, and 1.5 mg of CNT-OH and an appropriate amount of Nafion were added to obtain a mixed solution. Subsequently, the solution was stirred using a magnetic stirrer at 1000 r/min for 30 min, and then ultra-sonic dispersion was applied for 90 min to avoid the large aggregation of carbon nanotubes and to achieve average dispersion.

As shown in Figure 1d, a DP800 circuit board printer was used to create the electrodes. The cut PET film is placed on a printing platform with adsorption capability, and a program is run to spray the silver ink in the shape of the sensor circuit according to the designed electrode pattern. After printing, the substrate with the silver ink adhered to it is placed in a vacuum drying oven and baked at 150 °C for 20 min, then removed. The sensor mainly relies on the change in capacitance to the resonant frequency of the overall device to change. Therefore, the experiment is performed by pipetting a small amount of the prepared compound solution, maintaining the same height to titrate the solution uniformly to the position of the capacitance to form a uniform sensitive layer (Figure S3). In order to dry the surface substance, the device is placed in an oven and baked at 60 °C for 2 h to obtain the finished product.

## 3. Results and Discussion

### 3.1. Characterization

**Figure 2:**
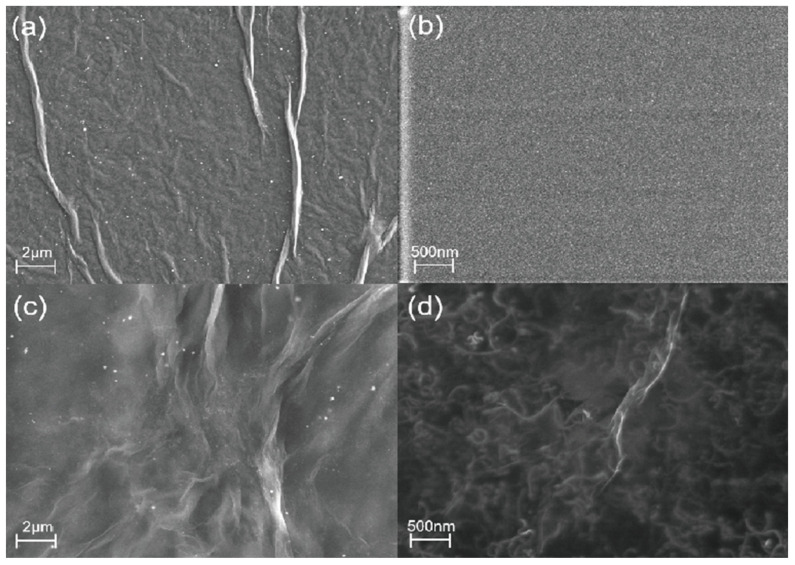
SEM images of (**a**) GO, (**b**) Nafion, (**c**) GO/Nafion, and (**d**) GO/CNT−OH/Nafion samples.

**Figure 3:**
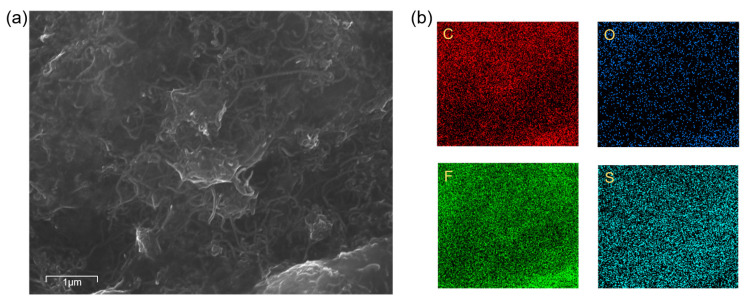
(**a**) SEM images of GO/CNT−OH/Nafion nanocomposites at 1 micron size; (**b**) element distribution of GO/CNT−OH/Nafion nanocomposites.

The morphological structure and element distribution of GO/CNT−OH/Nafion nanocomposites were characterized by scanning electron microscopy (SEM) and energy dispersive spectroscopy (EDS). Figure 2a shows the SEM image of GO monomers, which have an obvious wrinkled surface. In Figure 2b, the morphology of Nafion appears to be very flat. The folded state of the GO/Nafion composite in Figure 2c will be more obvious relative to Figure 2a. Figure 2d shows the SEM image of the GO/CNT−OH/Nafion nanocomposite. As shown in Figure 3a, MWCNTs OHs are well dispersed in the middle of graphite oxide nanosheets, supporting the distance between graphite oxide layers. From the element distribution in Figure 3b, it can be seen that the four elements C, O, F, and S are uniformly dispersed on the device surface, forming a uniform layer. The upper two figures in Figure 3b clearly show the distribution of C and O elements, confirming the presence of GO and MWCNT-OH. The S and F elements below in Figure 3b are uniformly distributed in the image, and both elements are provided by Nafion. Therefore, we can assume that Nafion is well dispersed on the material surface.

### 3.2. Humidity-Sensing Performance

**Figure 4:**
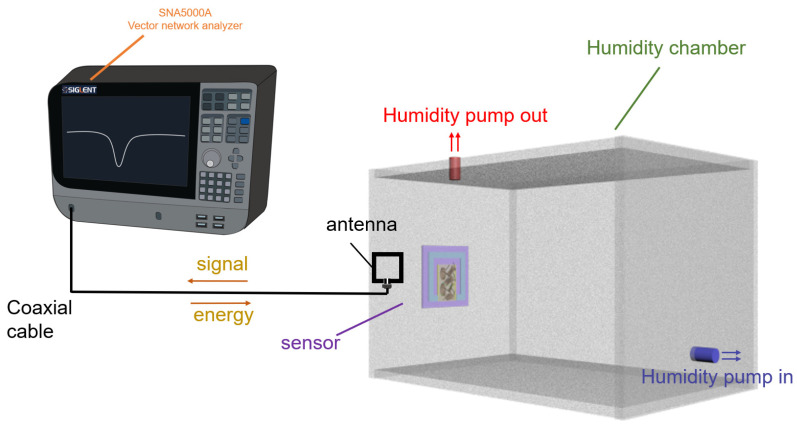
Test schematic diagram of sensor.

The experimental setup used for humidity sensing is shown in Figure 4. During the test, a Dingyang SNA5000A vector network analyzer was connected to the interrogating antenna through a coaxial cable, and the antenna was coupled wirelessly with the wireless humidity sensor placed on the inside wall of the humidity change box. The vector network analyzer obtained sensor signals at different humidity levels by transmitting sweeping signals from the antenna and receiving frequency signals from the sensor. In this paper, the frequency range of the VNA is 50 MHz to 250 MHz and all sensor test experiments are performed at 20 °C. The electromagnetic sweep signal is emitted by a transmitting coil connected to the network analyzer. As the sweep signal approaches the intrinsic frequency, the sensor’s inductor captures electromagnetic energy to activate the wireless sensor. The resonant frequency is obtained by extracting the minimum value of the resonance curve. In order to eliminate the possible effect of humidity on the inductance, we control the height of the pipette gun during drip coating such that the dripping solution does not spread to the inductor location. As a result, the *R_s_* and *L_s_* of the sensor remain essentially unchanged. If the relative humidity changes, the resonant frequency of the sensor changes due to the change in the dielectric constant (*ε*_s_). The humidity box is homemade, selected for a size of 33 cm × 23 cm × 21.5 cm, the interior is equipped with a Huayi pm6508 digital temperature and humidity meter and a commercial Xiaomi home temperature and humidity meter as the humidity measurement control standard. The interrogation coil in the experiment is a square coil with a side length of 39 mm prepared from copper wire with a cross-sectional diameter of 1 mm (Figure S2). In order to evaluate the humidity sensitivity performance of the sensor, the sensitivity S of the sensor is defined as:

(5) $$S\;(kHz/RH\%)=\frac{f_{a}-f_{b}}{H_{a}-H_{b}}$$

where f_a_ and f_b_ are the resonant frequencies of the sensor when the humidity is H_a_ and H_b_, respectively.

**Figure 5:**
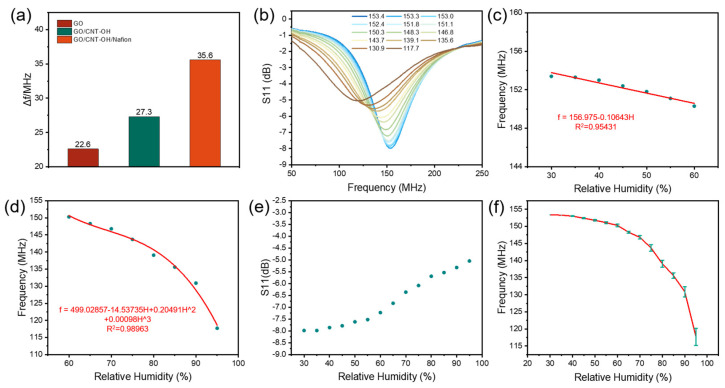
(**a**) Frequency shifts of the GO, CNT−OH/GO, and GO/CNT−OH/Nafion sensor; (**b**) S_11_−frequency results of the GO/CNT−OH/Nafion humidity sensor in 30–95% RH; frequency versus humidity curve of the GO/CNT−OH/Nafion humidity sensor (**c**) in a low humidity range (30–60% RH) and (**d**) in a high humidity range (60–95% RH); (**e**) signal level of GO/CNT−OH/Nafion humidity sensor in 30–95% RH; (**f**) image of resonant frequency with error bars as a function of humidity.

The total variation in the resonant frequency ∆f of wireless sensors deposited with the three materials of GO, GO/CNT−OH, and GO/CNT−OH/Nafion was tested at the same coil-to-device distance from 30% RH, with a rising gradient of 5% RH, up to 95% RH at room temperature. The results presented in Figure 5a indicate that the sensor coated with GO/CNT−OH/Nafion nanocomposites exhibited the highest frequency variation of 35.6 MHz, while the humidity sensors coated with GO and GO/CNT−OH exhibited lower frequency responses of 22.6 MHz and 27.3 MHz, respectively. In this regard, the humidity sensor coated with GO/CNT−OH/Nafion nanocomposites is more applicable and its sensing performance requires further exploration.

Figure 5b presents the *S*_11_ curves for different relative humidities in the range of 30–95% RH at room temperature. With a humidity increment of 5% RH, these curves indicate that the fabricated humidity sensor exhibits a frequency variation of 35.6 MHz in the range of 30–95% RH. In addition, Figure 5c,d, extracted from Figure 5b, show the frequency vs. humidity curves in the low humidity range (30–60% RH) and the high humidity range (60–95% RH), indicating that the resonant frequency of the humidity sensor manufactured decreases linearly with increasing humidity in the humidity range of 30–60% RH, with sensitivity and linearity R^2^ of 103 kHz/% RH and 0.95431. It can be seen that at the beginning of the test, the humidifier drums into the mist less, meaning there are fewer interactions between the sensor surface and water molecules; the beginning of the humidity brings about uneven changes, thus resulting in a low linearity in the early stage. However, the frequency-relative humidity curve in 60–95% RH matches the cubic curve very well with an excellent R^2^ of 0.98963 and a sensitivity up to 931 kHz/% RH, and the sensitivity value from 30–95% RH is 547 kHz/% RH. These results indicate that the fabricated humidity sensor can be used for accurate humidity monitoring in 30–95% RH. Furthermore, as shown in Figure 5e, all S11 values of each humidity point are greater than −5.04 dB in 30–95% RH, indicating that the overall monitoring difficulty is relatively small. Five sensors were made to test the resonant frequency error at different humidity levels, and it can be seen from Figure 5f that the sensor has a good repeatability. In addition, the humidity was reduced by dry argon gas, and the wet hysteresis line of the whole device was obtained after repeated measurements (Figure S1), indicating that the maximum hysteresis of the prepared humidity sensor is about 3% RH, which means that it can be used for accurate humidity measurements.

**Figure 6:**
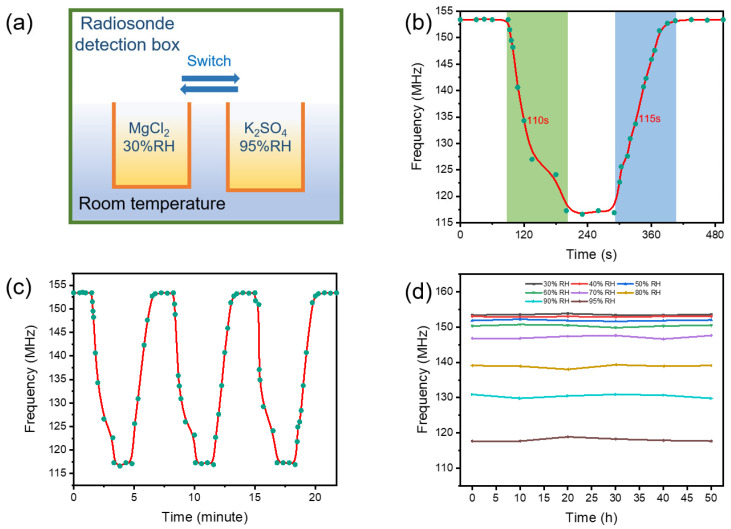
(**a**) The measurement appliance for response, recovery and repeatability tests; (**b**) the response and recovery, (**c**) repeatability, and (**d**) stability tests of the wireless GO/CNT−OH/Nafion humidity sensor.

In addition, the response, recovery, and repeatability of the humidity sensor were investigated. Two types of solutions (MgCl_2_ and K_2_SO_4_) were prepared and a test rig was constructed, as shown in Figure 6a. Due to the experimental conditions, the relative humidity of the saturated solutions of MgCl_2_ and K_2_SO_4_ was greater than 30% RH and 95% RH. We prepared a certain amount of these two solutions and adjusted the distance between the device and the mouth of the beaker to control the relative humidity of the two environments at 30% RH and 95% RH for stable testing. Then, the prepared sensor was brought to the top of the magnesium chloride solution until the resonant frequency of the sensor became stable. Subsequently, the sensor was quickly switched to the top of the K_2_SO_4_ solution. After the resonant frequency of the sensor had stabilized for about 90 s, the sensor was immediately switched back to the top of the MgCl_2_ solution until the resonant frequency stabilized. The response and recovery results presented in Figure 6b show that the response and recovery time of the sensor are 110 s and 115 s, respectively. The repeatability performance was probed by repeating similar response and recovery processes three times. The experimental results shown in Figure 6c reveal that the GO/CNT−OH/Nafion nanomaterials have a good repeatability in cycling and may be a repeatable sensing material for humidity detection. Finally, the necessary stability of the applicable sensors was investigated for 50 h at 30% RH, 40% RH, 50% RH, 60% RH, 70% RH, 80% RH, 90% RH, and 95% RH. Next, stability tests were performed on the prepared humidity sensors arranged in a test chamber at 30–95% RH. The frequencies of the same humidity sensors were recorded every 5 h for 50 h. As shown in Figure 6d, no acute frequency fluctuations were detected in the stability experiments, which confirmed that the GO/CNT−OH/Nafion composite nanomaterials are chemically stable to water molecules and the prepared humidity sensors possess a good stability.

### 3.3. Sensing Mechanism of the Humidity Sensor

**Figure 7:**
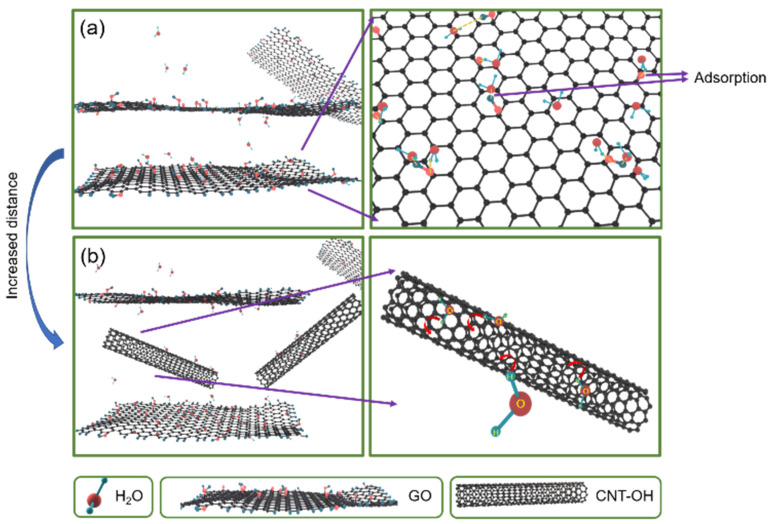
(**a**) Interaction between GO and water molecules; (**b**) interaction between MWCNTs/GO and water molecules.

As shown in Figure 7a, the coexistence of carbonyl, carboxyl, hydroxyl, and epoxy groups in GO ensures that GO has an excellent hydrophilicity to absorb water molecules [5]. However, when more GO layers are closely stacked, the distance between the layers decreases greatly, resulting in an unfavorable condition for the proliferation of water molecules. GO can adsorb pristine MWCNTs through π-stacking interactions, thus allowing the stable dispersion and fractionation of pristine MWCNTs in aqueous media. In Figure 7b, due to the hydroxylated MWCNTs used in this experiment, the presence of more hydroxyl groups enhances their binding to water molecules. When MWCNTs are dispersed in GO, they play a supporting role and cause larger ripples and more cavities in the GO/CNT−OH layer [34,38,39].

**Figure 8:**
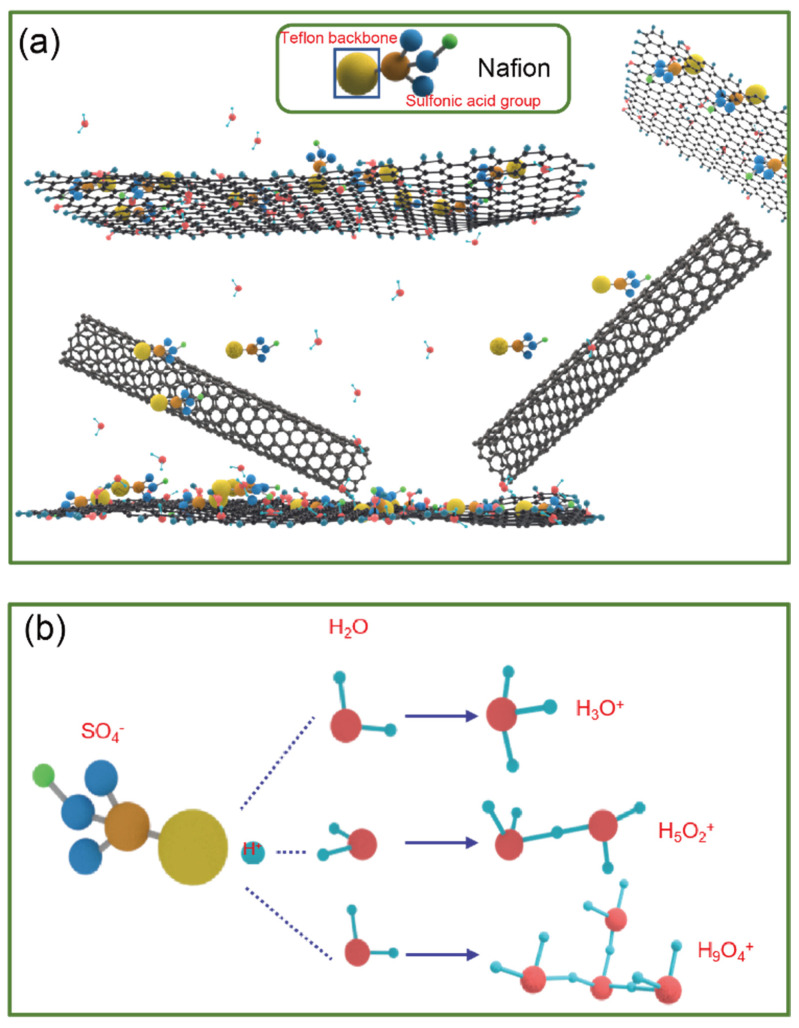
(**a**) Interaction between GO/MWCNT−OH/Nafion nanocomposites and water molecules; (**b**) dissociation of the sulfonic acid group of Nafion in water to create protons and hydrated protons.

As shown in Figure 8a, Nafion is a polystyrene sulfonic acid ionomer possessing superhydrophobic Teflon (tetrafluoroethylene) and hydrophilic sulfonic acid groups. The overall hydrophilic surface of the material increases due to the presence of MWCNTs that increase the distance between the GO layers. The hydrophilic ionic groups of SO_3_^−^ in Nafion absorb water from the air and will form ionic groups of H_3_O^+^, H_5_O_2_^+^, and H_9_O_4_^+^. The dispersion of GO sheets may mean that the hydrophobic region of GO sheets interacts with the hydrophobic Teflon backbone of Nafion, while the GO sheets and hydrophilic groups of Nafion are responsible for the adsorption of water molecules [40].

**Figure 9:**
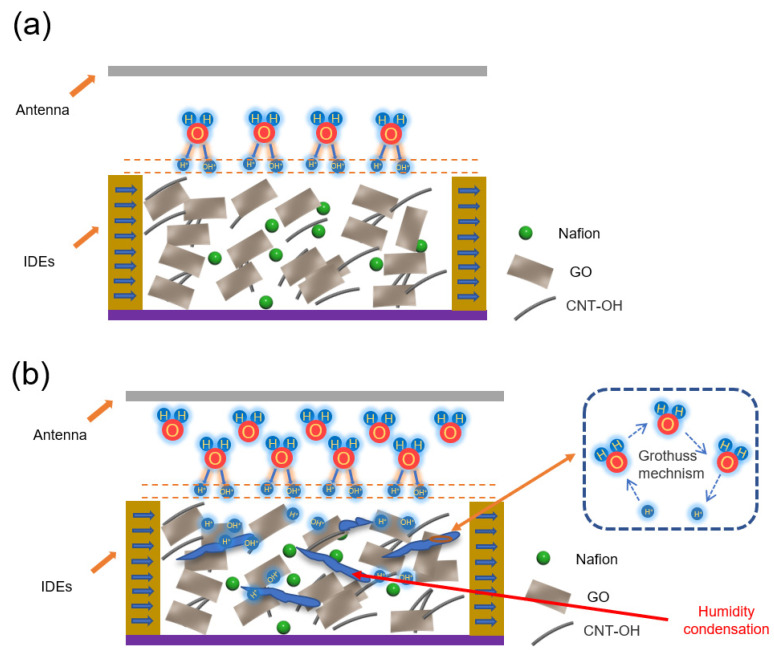
Sensing mechanism of humidity sensor (**a**) in a low humidity range (30–60% RH) and (**b**) a high humidity range (60–95% RH).

At the low humidity (30–60% RH) in Figure 9a, electrolytic conduction is triggered by the adhesion of water molecules to the material surface, leading to an increase in the relative permittivity, *ε_r_*, which indicates fluctuations in the corresponding capacitance and resonant frequency of the proposed sensor. The adsorption of water molecules on the surface is discontinuous, so the proton transfer is limited and occurs only in water clusters with discontinuous surfaces. Therefore, the electrodes have a weak permittivity drift, a small capacitive interference range, and a low sensitivity. At the low humidity (60–95% RH) in Figure 9b, considering that the supporting GO layer of carbon nanotubes and the Nafion will increase the overall hydrophilic surface area, when the humidity level continues to increase, the water molecules absorbed by the nanocomposites start to aggregate and condense into small droplets and even cluster into the interstices of the electrodes. It is worth noting that the presence of carbon nanotubes also promotes GO to trap water molecules. The same electrolytic conduction as in the low humidity range then expands and becomes continuous in the interstitial space of the IDE. More importantly, the Grottuss mechanism that amplifies the electrolytic conduction will occur significantly in condensed water. This enhancement occurs because this mechanism contributes to the transport of a large number of free protons in the condensate. As a result, the relative permittivity *ε_r_* greatly increases the sensitivity, which is much higher than that in the low humidity range (30–60% RH).

## 4. Conclusions

In summary, this paper presents an LC wireless passive humidity sensor based on GO/CNT−OH/Nafion nanocomposites. We observed the GO/CNT−OH/Nafion nanocomposites using SEM and fabricated the proposed humidity sensor by an inkjet printing technique. The experimental study shows that the proposed humidity sensor has a frequency variation of 103 kHz/%RH at low humidity (30–60% RH) and a sensitivity of 931 kHz/%RH at high humidity (60–95% RH), with a sensitivity value from 30–95% RH of 547 kHz/% RH. The response time and recovery time are 110 s and 115 s, respectively. In addition, the tests showed that the GO/CNT−OH/Nafion nanocomposite applied to the humidity sensor produced a maximum humidity hysteresis of about 3% RH at 30–95% RH, the resonant frequency remained basically unchanged after 50 h of testing, and the whole sensor possessed a good stability. After conducting several repeated experiments, it was found that the resonant frequency error of the whole sensor was low and did not affect the overall sensing test, which proved the reproducible preparation of the sensor. Finally, the humidity-sensing mechanism of the proposed sensor was analyzed in this paper, and it was found that GO enhanced the hygroscopic properties of GO/CNT−OH/Nafion nanocomposite when it was supported by MWCNT-OH and included uniformly dispersed Nafion. Therefore, our proposed humidity sensor is suitable for humidity detection above 30% RH in both sealed and open environments.

## Figures and Tables

**Table 1:** Performance comparison analysis of our work and certain previous work.

Sensing Material	Range (% RH)	Response (s)	Recovery (s)	Sensitivity	Reference
DuPont 5018	20–90	-	>1800	1.1 kHz/%RH	[22]
PEL	30–80	240	360	371 kHz/%RH	[23]
Paper	11–97	3600	2400	140 kHz/%RH	[24]
GO/CNT−OH/Nafion	30–95	110	115	547kHz/%RH	This work

**Table 2:** Detailed parameters of the inkjet-printed device.

Symbol	Unit (mm)
*L*	40
*W*	5
*w*_g_	0.25
*g*	0.25
*g*_e_	0.25
*l*_c_	17.75
*h*	0.002

## Data Availability

All available data is contained within the article.

## Supplementary Materials

The following supporting information can be downloaded at: https://www.mdpi.com/article/10.3390/nano13131925/s1, Figure S1: Hysteresis results of as-prepared humidity sensor; Figure S2: Vector network analyzer with interrogation coil; Figure S3: Cross section view of sensor sensitive layer.
