# Supplementary material for: GO/CNT−OH/Nafion Nanocomposite Humidity Sensor Based on the LC Wireless Method
Source: Nanomaterials (Basel). 2023 Jun 24;13(13):1925. doi: 10.3390/nano13131925 (PMC10343782; doi:10.3390/nano13131925)
Supplement: Supplementary file 1 [file nanomaterials-13-01925-s001.zip › nanomaterials-2445009-supplementary.pdf]

Supplementary Materials

GO/CNT−OH/Nafion Nanocomposite Humidity Sensor Based on the LC Wireless Method

Chengkai Wang, Chunxiao Jiao, Meng Wang, Jinghong Pan and Qi Wang *

College of Sciences, Northeastern University, Shenyang 110819, China; 2100178@stu.neu.edu.cn (C.W.); jiaochunxiao@stumail.neu.edu.cn (C.J.); winnie@stumail.neu.edu.cn (M.W.); 2100169@stu.neu.edu.cn (J.P.)

*   Correspondence: wangqi@mail.neu.edu.cn

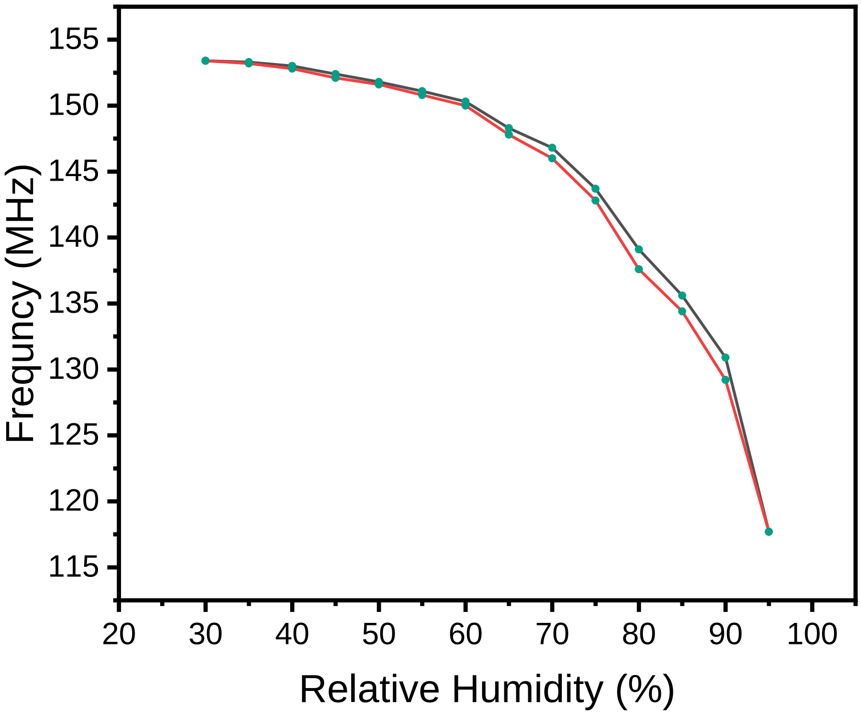

Figure S1. hysteresis results of as-prepared humidity sensor.

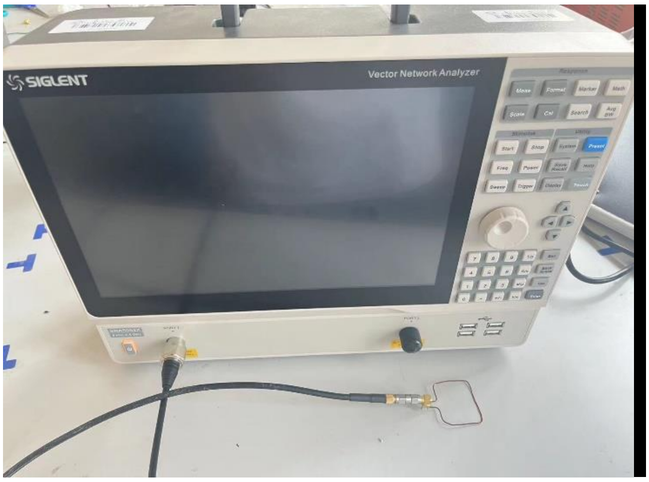

Figure S2. Vector network analyzer with interrogation coil.

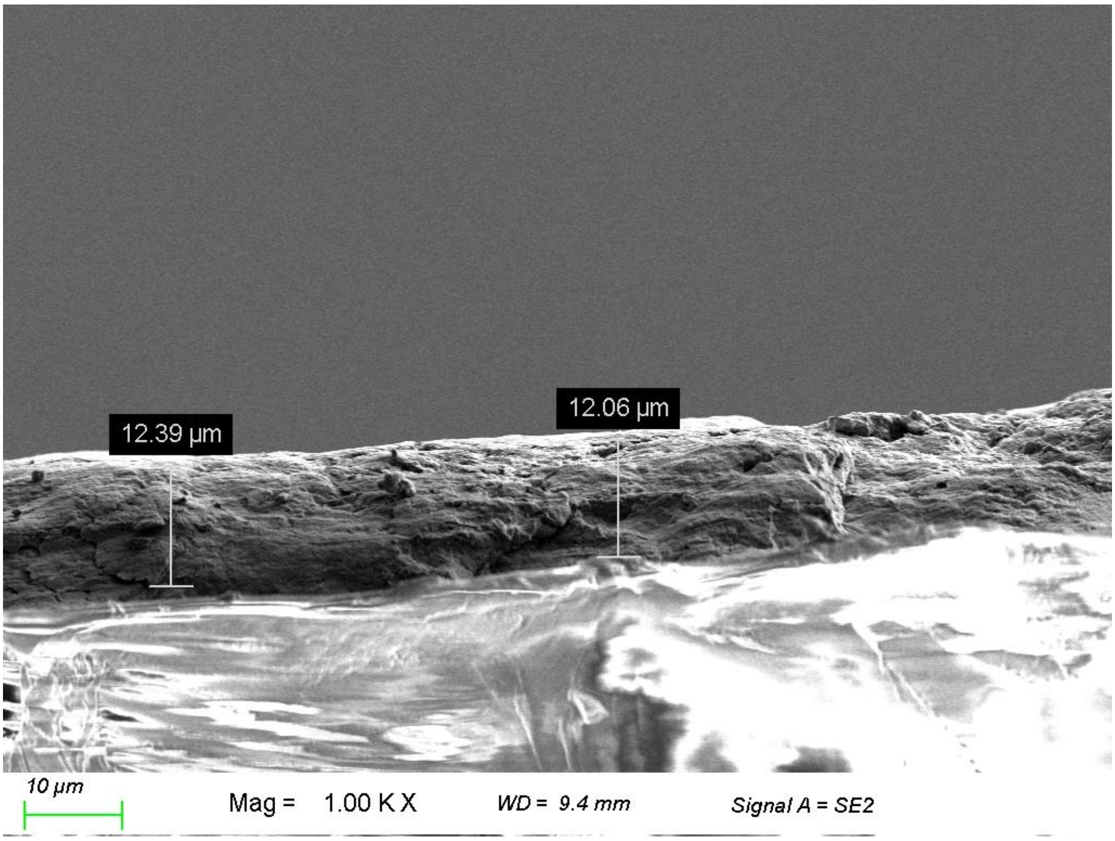

Figure S3. Cross section view of sensor sensitive layer.
